# Low-Normal Thyroid Function and Novel Cardiometabolic Biomarkers

**DOI:** 10.3390/nu7021352

**Published:** 2015-02-16

**Authors:** Lynnda J.N. van Tienhoven-Wind, Robin P.F. Dullaart

**Affiliations:** Department of Endocrinology, University of Groningen and University Medical Center Groningen, Groningen, AV Groningen 19713, the Netherlands; E-Mail: l.j.n.van.tienhoven-wind@umcg.nl

**Keywords:** bilirubin, biomarkers, cardiovascular disease, cholesteryl ester transfer, dyslipidemia, lipids, metabolic syndrome, non-alcoholic fatty liver disease, subclinical hypothyroidism, thyroid hormones

## Abstract

The concept is emerging that low-normal thyroid function, *i.e.*, either higher thyroid-stimulating hormone or lower free thyroxine levels within the euthyroid reference range, could contribute to the development of atherosclerotic cardiovascular disease. It is possible that adverse effects of low-normal thyroid function on cardiovascular outcome may be particularly relevant for specific populations, such as younger people and subjects with high cardiovascular risk. Low-normal thyroid function probably relates to modest increases in plasma total cholesterol, low density lipoprotein cholesterol, triglycerides and insulin resistance, but effects on high density lipoprotein (HDL) cholesterol and non-alcoholic fatty liver disease are inconsistent. Low-normal thyroid function may enhance plasma cholesteryl ester transfer, and contribute to an impaired ability of HDL to inhibit oxidative modification of LDL, reflecting pro-atherogenic alterations in lipoprotein metabolism and HDL function, respectively. Low-normal thyroid function also confers lower levels of bilirubin, a strong natural anti-oxidant. Remarkably, all these effects of low-normal thyroid functional status appear to be more outspoken in the context of chronic hyperglycemia and/or insulin resistance. Collectively, these data support the concept that low-normal thyroid function may adversely affect several processes which conceivably contribute to the pathogenesis of atherosclerotic cardiovascular disease, beyond effects on conventional lipoprotein measures.

## 1. Introduction

It is widely appreciated that overt hypothyroidism adversely affects cardiovascular morbidity and mortality [1,2]. However, it has not been unequivocally established whether subclinical hypothyroidism (SCH) also contributes to the development of atherosclerotic cardiovascular disease (CVD) [3]. The prevalence of overt hypothyroidism varies from 0.1% to 3.7% [4,5,6]. SCH, defined as a plasma thyroid-stimulating hormone (TSH) level above the institutional reference range (in most laboratories > 4.0−4.5 mU·L^−1^) combined with a plasma free thyroxine (FT4) or free triiodothyronine (FT3) level within the reference range [3], is more common, ranging between 4.6% and 8.5% in the general population, even rising to 15% in elderly populations [5,6].

The high prevalence of thyroid dysfunction has considerable consequences for a several important health issues, including cardiometabolic disorders [7]. Since each individual probably has a narrow set-point of thyroid function status [8], the concept is now emerging that low-normal thyroid, *i.e.*, higher TSH and/or lower free thyroid hormone levels within the euthyroid reference range, even when determined at a single time-point, could have a negative impact on atherosclerotic cardiovascular disease [7,9]. Of interest is a recent systematic review comprising observational studies, which has suggested an increased risk of higher plasma total cholesterol, low density lipoprotein (LDL) cholesterol and triglycerides, as well as obesity, metabolic syndrome and chronic kidney disease in the context of low-normal thyroid function [7].

This review is focused on recent data with respect to the effect of low-normal thyroid function on (surrogate markers of) cardiovascular outcome, as well as on novel lipid and non-lipid biomarkers which are conceivably involved in the pathogenesis of atherosclerotic CVD.

### 1.1. Subclinical Hypothyroidism, Low-Normal Thyroid Function and Atherosclerotic Cardiovascular Disease

The extent to which subclinical hypothyroidism is causally implicated in accelerated development of atherosclerosis has not been unequivocally established. A considerable number of cross-sectional and prospective studies on the association between SCH and cardiovascular disease have been reported during the past two decades [3,10,11,12,13,14,15]. In an early report from the Rotterdam cohort, an elevated age-adjusted prevalence of aortic atherosclerosis and myocardial infarction was documented among 1149 postmenopausal women [10]. These associations remained significant after an additional adjustment for the body mass index (BMI), total cholesterol and high density lipoprotein (HDL) cholesterol, blood pressure and smoking status, and were slightly stronger in women who also had antibodies to thyroid peroxidase. In this cross-sectional study, the population attributable risk of SCH for myocardial infarction was as large as that of established cardiovascular risk factors [10]. On the other hand, in a community-dwelling population study from the USA, comprising 3233 subjects, SCH did not independently predict cardiovascular morbidity and mortality during a follow-up period of about 12 years [11]. Remarkably, a large retrospective study from Denmark, which included more than 500,000 subjects, recently demonstrated a modest reduction in all-cause mortality associated with SCH during 5.5 years of follow-up (hazard ratio, 0.92) [12]. The association of a lower all-cause mortality with SCH was observed only in subjects aged > 65 years. In that report, the occurrence of major adverse cardiovascular events was unrelated to SCH [12].

Three meta-analyses have been published, which aimed at assessing the strength of the association between SCH and CVD. Ochs *et al*. reported that SCH is associated with a non-significant 20% higher relative risk for cardiovascular disease in a meta-analysis involving a total of 14,449 participants [13]. In secondary analysis, cardiovascular mortality risk appeared to be greater in those studies in which mean age of study participants was <65 years compared to studies in which mean age was ≥65 years (relative risk, 1.50 *vs.* 1.20) [13]. Razvi *et al*. observed a modestly increased prevalence of ischemic heart disease attributable to SCH (odds ratio, 1.23; 27,267 subjects); the incidence of CVD associated with SCH was not significant (odds ratio, 1.27; 9627 subjects) [14]. In line with the first meta-analysis, they reported an increased prevalence and incidence of ischemic heart disease in conjunction with SCH in studies including subjects <65 years of age (odds ratio, 1.57 and 1.68, respectively), but not in studies which only included subjects ≥65 years of age (odds ratios, 1.01 and 1.02, respectively) [14]. A similar age-dependent trend was found for cardiovascular mortality [14]. A third meta-analysis by Singh *et al*. documented that SCH confers an increased relative risk of both prevalent coronary heart disease (relative risk, 1.53; 11,495 subjects), and incident coronary heart disease (CHD) (relative risk, 1.19; 7026 subjects [15]. They also observed an elevated risk of cardiovascular death among SCH subjects (relative risk, 1.28), although not significantly so in all-cause mortality (relative risk, 1.35) [15]. Collectively, several reports have thus suggested that the association of SCH with higher risk of CVD may be age-dependent, without any excess risk or perhaps even protection in elderly subjects [3,12,13,14]. Given the observational nature of these studies, it should be noted that neither the possibility of residual bias, nor that of reverse causation, can be fully excluded.

Carotid intima–media thickness (cIMT), measured by ultrasound, is a predictor of coronary heart disease and stroke, and represents an established surrogate marker of subclinical atherosclerosis [16,17,18]*.* cIMT has been found to be greater in subjects with overt hypothyroidism compared to euthyroid subjects, and may decrease after levothyroxine replacement [19]. In marked contrast, a German study that included subjects older than 45 years across the range from hypothyroidism to hyperthyroidism showed that cIMT was lower in the context of higher TSH levels [20]. In another cross-sectional study, no significant relation of cIMT with TSH was found after adjustment for conventional cardiovascular risk factors [21]. However, in a meta-analysis of observational studies including 3602 participants, Gao *et al*. recently demonstrated that SCH is associated with increased cIMT, particularly in the context of TSH levels > 10 mU·L^−1^ [22]. This report showed that SCH is associated with higher systolic blood pressure, higher plasma total cholesterol, low density lipoprotein (LDL) cholesterol and triglycerides [22]. It remains to be established in how far greater cIMT in SCH is attributable to these cardiovascular risk factors. Nonetheless, this meta-analysis is in agreement with the supposition that SCH may confer increased risk of subclinical atherosclerosis. In addition, SCH was found to be associated with subclinical atherosclerosis, using coronary artery calcification as marker of subclinical atherosclerosis, but only in subjects with concomitant non-alcoholic fatty liver disease (NAFLD) [23].

At present, less information is available about the association o low-normal thyroid function with cardiovascular outcome. The Hunt Study prospectively examined the association of CHD mortality with TSH levels within the reference range in a Norwegian population-based cohort study including 25,313 men and women without known thyroid disease, cardiovascular disorders or diabetes mellitus at baseline [24]. In this study, women with higher TSH levels within the normal range were positively and linearly associated with mortality from CHD (hazard ratio upper *vs.* lower tertile, 1.69). This association remained significant after adjustment for conventional cardiovascular risk factors, and was also only present in non-smokers. In non-smoking men, there was a positive but non-significant association of CHD mortality with TSH levels within the reference range. These important findings suggest that low-normal thyroid function, as assessed by a high-normal TSH level, may increase the risk of CVD [24]. On the other hand, in another prospective study from the UK, 1191 individuals did not show a significant relationship between (cardiovascular) mortality and variation in TSH levels within the reference range, but it should be taken into consideration that this survey was primarily focused on subclinical hyperthyroidism [25].

Several studies have appeared during the past few years which were specifically aimed at evaluating whether cIMT is also influenced by low-normal thyroid function. In a cross-sectional study including 78 Caucasian non-smoking, middle-aged, strictly euthyroid subjects, cIMT was found to be inversely related FT4, independent of plasma lipids and BMI [26]. In agreement, cIMT was associated inversely with FT4 among 643 euthyroid Japanese subjects [27]. However, cIMT was found to be unrelated to thyroid function parameters among middle-aged women, despite a positive association of TSH with pulse wave velocity [28]. Altogether, these results would agree with the hypothesis that low-normal thyroid function may promote the development of atherosclerosis. Clearly, large-scale prospective studies both with incident cardiovascular events and with cIMT changes over time as outcome are required to more definitely test whether variations in thyroid function within the normal range indeed confer increased cardiovascular risk.

### 1.2. Changes in Plasma Lipoproteins and C-Reactive Protein Consequent to Subclinical Hypothyroidism and Low-Normal Thyroid Function

The effects of SCH on plasma lipoprotein levels have been examined in a considerable number of studies, as reviewed elsewhere [29,30,31,32,33]. In summary, SCH is likely to result in modest increases in plasma total cholesterol, LDL cholesterol, triglycerides and apolipoprotein (apo) B levels (Table 1). In SCH, these lipoprotein abnormalities may at least in part be normalized after levothyroxine treatment (Table 1). Minor and inconsistent changes in HDL cholesterol and in apoA-I, its most abundant apolipoprotein, have been reported in SCH [33]. Thus, taking into account that HDL cholesterol may be considerably elevated in overt hypothyroidism [30,33,34], it is remarkable that there are only minor elevations or even lower HDL cholesterol levels in conjunction with SCH. In addition, plasma levels of lipoprotein (a) (Lp(a)), a pro-atherogenic subfraction of LDL that is formed by disulfide bridges between apoB and apo(a), is probably unchanged in SHC [33], despite robust increases in overt hypothyroidism [35].

The effect of low-normal thyroid function on plasma (apo)lipoproteins has been evaluated by several studies so far. Nine larger studies (each comprising > 500 individuals; 90,041 subjects in total) have been reported in which the relationship of lipoprotein variables with high-normal TSH, low-normal FT4 and/or low-normal FT3 levels was determined [24,27,36,37,38,39,40,41,42]. As shown in Table 2, a positive relationship of plasma total cholesterol, LDL cholesterol and triglycerides was found in 3,1 and 3 of these reports, respectively. Four studies did not show a significant relationship of plasma total cholesterol and LDL cholesterol with TSH, whereas in two studies the relationship with triglycerides was not significant. The relationship of these lipoprotein measures with FT4 was assessed in four studies. Plasma total cholesterol and LDL cholesterol were positively related to FT4 in one study but inversely in another report. Higher plasma triglycerides were related to lower FT4 levels in two studies and to higher FT4 in one paper. Variable effects of low-normal thyroid function on HDL cholesterol were demonstrated. In all these reports, the relationship of lipoprotein measures with TSH and/or FT4 were rather low (correlation coefficients < 0.12). Taken together, these studies suggests that low-normal thyroid function may give rise to small increases in plasma levels of apoB-containing lipoproteins, in keeping with qualitatively comparable lipoprotein alterations in SCH.

**Table 1:** Effects of overt and subclinical hypothyroidism on plasma (apo) lipoproteins, and of levothyroxine treatment in subclinical hypothyroidism.

	Overt hypothyroidism	Subclinical hypothyroidism	Levothyroxine treatment
Total cholesterol	↑	↑, ns	↓, ns
LDL cholesterol	↑	↑, ns	↓, ns
HDL cholesterol	↑	↓, ns	↑,ns
Triglycerides	↑	↑, ns	↓, ns
Apolipoprotein B	↑	↑	↓
Apolipoprotein A-I	↑	ns	ns
Lp(a)	↑	ns	ns

HDL: high density lipoproteins; LDL: low density lipoproteins; Lp(a): lipoprotein (a). ↑: increased; ↓: decreased; ns: no significant effect.

**Table 2:** Relationships of plasma (apo) lipoproteins with thyroid function parameters in euthyroid subjects as determined by cross-sectional analyses in population-based studies (all studies included > 500 individuals).

Reference	*N*	Analysis	Total cholesterol	LDL cholesterol	HDL cholesterol	Triglycerides	apoB	apoA-I
Asvold [24]; Year 2007	27,727	Men and women separately Adjusted for age, smoking and prandial state	TSH: +	TSH: +	TSH: -	TSH: +		
Roos [36]; Year 2007	1581	Men and women combined Crude	TSH: *ns* FT4: - FT3: -	TSH: *ns* FT4: - FT3: -	TSH: + FT4: *ns* FT3: *ns*	TSH: + FT4: - FT3: -	TSH: *ns* FT4: *ns* FT3:-	TSH: + FT4: *ns* FT3: *ns*
Takamura [27]; Year 2009	643	Men and women combined Crude	TSH: *ns* FT4: *ns*	TSH: *ns* FT4: *ns*	TSH: - FT4: *ns*	TSH: *ns* FT4: +		
Kim [37]; Year 2009	44,196	Men and women separately Crude	FT4: +	FT4: +	FT4: +	FT4: -		
Park [38]; Year 2009	949	Postmenopausal women Crude	TSH: +	TSH: +	TSH: ns	TSH: +		
Garduño-Garcia [39]; Year 2010	2771	Men and women combined Adjusted for age, sex and BMI	TSH: + FT4: *ns*	TSH: *ns* FT4: *ns*	TSH: *ns* FT4: +	TSH: + FT4: *ns*		
Lee [40]; Year 2011	7270	Men and women combined Adjusted for age, sex, BMI, season, menopausal status	TSH: +	TSH: +	TSH: *ns*	TSH: +		
Lu [41]; Year 2011	1240	Men and women combined Crude	TSH: *ns*	TSH: *ns*	TSH: *ns*	TSH: *ns*		
Wang [42]; Year 2012	3664	Men and women combined Crude	TSH: *ns*	TSH: *ns*	TSH: +	TSH: *ns*		

BMI: body mass index; FT4: free thyroxine; FT3: free triiodothyronine; TSH: thyroid-stimulating hormone; *ns*: not significant; Positive (+) and negative (-) associations are indicated.

As reviewed elsewhere, inconsistent associations between SCH and enhanced low-grade chronic inflammation, most commonly assessed by high sensitive C-reactive protein (hsCRP) levels, have been reported so far [3]. In addition, besides a lack of difference in hsCRP, there was no difference in homocysteine levels between SCH and euthyroid subjects in the National Health and Nutrition Examination Survey [43]. Moreover, there was no association of hsCRP with low-normal thyroid function in two reports on the association of cIMT with low-normal thyroid function [26,27]. Thus, it seems unlikely that low-normal thyroid function has a major influence on low-grade chronic inflammation.

## 2. Regulation of Lipid Homeostasis and Lipoprotein Metabolism by Thyroid Hormones

Thyroid hormones have multifaceted and crucial roles in intracellular lipid homeostasis, as well as in plasma lipoprotein metabolism [30,31,33,44].

### 2.1. Cholesterol Homeostasis

Thyroid hormones induce the expression of 3-hydroxy-3-methylglutaryl coenzyme A (HMG-CoA) reductase, a key regulator of cholesterol synthesis, which is able to convert HMG-CoA to mevalonate [45]. LDL receptor expression is also stimulated by thyroid hormones. The LDL receptor gene and one of its main regulatory factors, sterol regulatory element-binding protein-2 (SREBP-2), contain a thyroid hormone responsive element [46,47]. Consequently, LDL clearance is increased by the action of thyroid hormones. This results in lower plasma LDL cholesterol in hyperthyroidism and higher levels in hypothyroidism, despite stimulating effects of thyroid hormone on hepatic cholesterol synthesis [30,31,33]. During the past few years it has become increasingly clear that the proprotein converstase subtilisn-kexin type 9 (PCSK9) pathway is intricately involved LDL metabolism [48,49]. PCSK9 is a secreted protease that binds to the extracellular domain of the LDL receptor, thereby targeting it for lysosomal degradation after endocytosis. PCSK9 thus prevents LDL receptor recycling to the cell surface, impairing LDL receptor abundancy. Plasma PCSK9 levels are likely to be physiologically relevant, because LDL clearance is decreased at higher PCSK9 plasma levels [50]. Remarkably, PCSK9 expression is also regulated by SREBP-2 [48,49], again indicating the crucial role of this transcriptional factor in cellular cholesterol homeostasis. Against this background we recently determined whether low-normal thyroid function, as indicated by high-normal TSH levels, may confer increased PCSK9 plasma levels [51]. In non-obese subjects, plasma PCSK9 concentrations were related positively with TSH levels within the euthyroid range [51]. This finding raises the possibility that thyroid function status is a determinant of cellular cholesterol trafficking by affecting LDL receptor expression via PCSK9 regulation. However, whether the plasma concentration of PCSK9 is a useful marker of incident CVD awaits further study [52]. Of further note, while it is generally believed that effects of thyroid function status are exerted through intracellular actions of thyroid hormones, there is also recent evidence which suggests that TSH could have a direct effect on HMG-CoA expression [53]. Finally, biliary excretion of cholesterol and neutral steroids is decreased, whereas intestinal cholesterol absorption is increased in hypothyroidism [54].

### 2.2. Triglyceride Homeostasis

Thyroid hormones have the ability to increase the mobilization of stored triglycerides by stimulating adipose tissue lipolysis [55], although there remain areas of uncertainty regarding the interplay between insulin action and adipose tissue lipolysis in hypothyroidism [56]. Circulating free fatty acid and glycerol levels are increased in hyperthyroidism [57,58], which results in an increased delivery of free fatty acids to the liver for subsequent re-esterification to triglycerides [55]. Notably, thyroid hormones concurrently stimulate hepatic fatty acid β-oxidation as well [55]. As a result of these divergent actions, hypothyroidism most likely results in enhanced hepatic triglyceride accumulation [9,59,60]. Hepatic fat accumulation is considered to represent a main driving force responsible for increased production of large very-low-density lipoprotein (VLDL) particles in the metabolic syndrome (MetS), as well as in Type 2 diabetes mellitus (T2DM) [61,62,63]. Furthermore, it should be noted that the clearance of VLDL particles in the circulation is decreased in hypothyroidism, due to impaired activity of lipoprotein lipase, resulting in decreased lipolysis of VLDL-derived triglycerides [57,58], and probably also to diminished hepatic VLDL removal via LDL receptor-related protein 1 [64]. Against this background, we set out to determine the relationship of VLDL subfractions with low-normal thyroid function. In a mixed group of euthyroid T2DM and non-diabetic subjects, lower FT4 levels were found to predict increased an concentration of large VLDL particles and a greater VLDL size independent of the presence of T2DM [65]. This finding is consistent with the hypothesis that abnormalities in triglyceride metabolism may represent an early event in the setting of low-normal thyroid function.

### 2.3. Plasma lipoprotein metabolism

The pathophysiological mechanisms responsible for increased plasma levels of LDL cholesterol and triglycerides in overt hypothyroidism, and opposite changes in overt hyperthyroidism have been detailed in the previous section [30,31,33]. Thyroid hormones also affect Lp(a) regulation [30,31,33,35,66]. The responsible mechanisms are not precisely known but probably involve LDL receptor-mediated clearance, as indirectly supported by recent observations which show that Lp(a) is dose-dependently decreased in response to administration of PCSK9 inhibiting monoclonal antibodies [67].

Thyroid hormones are prominently involved in the regulation of a number of other factors that play crucial roles in HDL metabolism, *i.e*., lecithin:cholesterol acyltransferase (LCAT), cholesteryl ester transfer protein (CETP), and hepatic lipase [30,31,33,35,68,69]. The HDL-associated enzyme, LCAT, is able to esterify free cholesterol to cholesteryl esters, thereby enhancing the conversion of lipid-poor pre β-HDL particles to larger, spherical HDL particles [70]. Subsequently, HDL-derived cholesteryl esters are transferred to triglyceride-rich lipoproteins by the action of CETP. As a consequence of this CETP-mediated cholesteryl ester transfer (CET) process, the cholesterol content of HDL is decreased [70,71,72]. During the CET process triglycerides are transferred in the opposite direction from triglyceride-rich lipoproteins to HDL, resulting in triglyceride-enriched HDL particles. Triglycerides in these HDL particles are then hydrolyzed by hepatic lipase giving rise to smaller-sized HDL particles [70,71]. By a comparable mechanism, the CET process also contributes to the generation of atherogenic small-dense LDL particles. The plasma activities of LCAT, CETP and hepatic lipase are all increased by thyroid hormones [34,68,69,73]. Alterations in these factors act in concert to increase HDL cholesterol and HDL size in the context of severe hypothyroidism, with opposite HDL changes in overt hyperthyroidism [30,31,33,73].

### 2.4. Novel Lipid Biomarkers and Low-Normal Thyroid Function

It is beyond doubt that low plasma levels of HDL cholesterol predict the future development of CVD [74,75], but the validity of the concept that raising HDL cholesterol as such provides a meaningful approach to reduce CVD risk has been seriously challenged during the past few years [76,77,78].

As outlined above, the CET process contributes to an unfavorable plasma lipoprotein profile by lowering cholesterol content in HDL particles, raising the cholesterol content in triglyceride-rich lipoproteins, and providing a pathway responsible for the generation of small dense LDL particles. Remarkably, plasma CETP mass or activity levels *per se* are unlikely to predict incident CVD [79,80,81]. On the other hand, plasma CET, assayed by an isotope method that reflects CETP-mediated transfer of cholesteryl esters from endogenous HDL to VLDL and LDL, associates with increased cIMT [82], prospectively predicts CVD, even independent of plasma CETP mass and lipoprotein concentrations [83], and relates to younger age at presentation of myocardial infarction [84]. In view of the potential relevance of plasma CET for the development of atherosclerosis, and considering that plasma CET is elevated in T2DM in conjunction with high triglycerides [70,82], we have recently evaluated whether plasma CET is enhanced in the context of low-normal thyroid function. In a study comprising euthyroid subjects with and without T2DM, it was documented that plasma CET is positively related to high-normal TSH levels in T2DM subjects, but not in non-diabetic individuals [85]. It was, furthermore, shown that plasma CET was modified in the context of chronic hyperglycemia, as evidenced by a positive interaction of TSH with the presence of T2DM, or alternatively with fasting plasma glucose and the glycated hemoglobin level on plasma CET. Additionally, the positive relationship of plasma CET with triglycerides in T2DM subjects was more outspoken with higher TSH levels [85]. Collectively, these data underscore the concept that low-normal thyroid function may adversely influence a pro-atherogenic lipid biomarker in conjunction with chronic hyperglycemia and hypertriglyceridemia. From these results it seems conceivable that strict control of thyroid function status, as guided by a TSH level in the low-normal range, could be advantageous in T2DM patients who require levothyroxine substitution therapy.

The relevance of HDL functional properties for cardioprotection has been recently emphasized [86,87]. In agreement with the contention that HDL may become dysfunctional, it was shown that cardiovascular risk is increased in subjects with concurrently high levels of HDL cholesterol and C-reactive protein [88]. Moreover, HDL’s anti-inflammatory function is diminished in acute myocardial infarction [89], and may predict recurrent coronary events, even independent from HDL cholesterol and plasma apoA-I [90]. Besides other potentially relevant athero-protective functions, HDL inhibits LDL from oxidative modification, thereby protecting against oxidative stress [87,89,91]. Importantly, it was demonstrated previously that LDL oxidizability is increased in overt hypothyroidism [92,93]. Additionally, oxidative stress markers are elevated in SCH [94,95], whereas high-normal TSH levels within the euthyroid range associate with elevations in oxidized LDL levels [96]. Oxidative stress is known to be enhanced in T2DM [81,97,98]. We, therefore, tested the extent to which low-normal thyroid function impacts on the ability of HDL to inhibit LDL oxidation in euthyroid subjects with varying degrees of glucose intolerance [90]. In this study, we used inhibition of HDL (standardized for the amount of HDL cholesterol) on 2,2′-azobis (2-methylproprionamidine) dihydrochloride (AAPH)-induced formation of thiobarbituric acid reactive substances (TBARS) as read-out. It was found that an impaired HDL anti-oxidative capacity was determined by low-normal FT4 levels in T2DM subjects, but not in subjects with normal fasting plasma glucose [99]. We also showed that the relationship of FT4 with HDL anti-oxidative capacity varied according to glucose tolerance status. These findings agree with the concept that low-normal thyroid function may negatively impact on in this metric of HDL function under circumstances of chronic hyperglycemia. Thus, it appears that T2DM subjects may be particularly susceptible to low-normal thyroid function-related adverse effects on several lipid biomarkers which conceivably play a role in the pathogenesis of atherosclerotic CVD.

## 3. Subclinical Hypothyroidism, Low-Normal Thyroid Function and Metabolic Syndrome

The metabolic syndrome (MetS), commonly defined according to the NCEP-ATPIII criteria, is a cluster of metabolic disorders which include central obesity, elevated plasma glucose, high triglycerides, low HDL cholesterol and hypertension [100,101].

The association of the presence of MetS with low-normal thyroid function has been documented in a number of epidemiological surveys [36,37,38,39,102]. In a cross-sectional analysis involving non-diabetic subjects participating in the PREVEND (Prevention of Renal and Vascular End stage Disease) study, low-normal thyroid function was predictive of 4 of the 5 MetS components; only blood pressure was unrelated to TSH and FT4 [34]. A high-normal TSH level also determined an increased prevalence of MetS in the Healthy ABC study, but did not predict new-onset MetS during follow-up [102]. The presence of MetS was associated with low-normal FT4 levels in Korean men and women [37], and in post-menopausal women [38]. In addition, more severe insulin resistance, as determined by homeostasis model assessment, was associated with low-normal FT4 levels in the PREVEND cohort [36] and a Mexican population-based study [39]. However, neither the prevalence of MetS, nor its individual components, were related to SCH in the Mexican study [39]. Additionally, SHH was found to predict the presence of MetS and more severe insulin resistance only in subjects with concomitant NAFLD [23].

A considerable number of studies have dealt with the relationship of thyroid function with obesity, as reviewed elsewhere [103]. Thyroid hormones increase resting energy expenditure [104], which generally leads to weight loss in overt hyperthyroidism, but the increase in body weight in overt hypothyroidism seems to be mainly due to fluid retention [103]. Nonetheless, small differences in thyroid function even within the normal range are likely to be relevant for obesity, as judged by a positive association of BMI with the TSH level and an inverse association with FT4, as well as by a higher prevalence of obesity in a community-dwelling study among adults from Denmark [105]. Likewise, a positive association of BMI and waist circumference with TSH was observed in adult men and women participating in the National Health and Nutrition Examination Survey [106]. On the other hand, the hypothalamic-pituitary-thyroid axis is probably activated in obesity [103], which could in part be attributable to interactions with adipokines, such as leptin [107]. Indeed, TSH, as well as FT4 and FT3 levels, may all be higher in obese children [108].

Hypothyroidism leads to insulin resistance in striated muscle and adipose tissue, which may be due at least in part to decreased translocation of GLUT4 to the cell membrane, impairing glucose transport [56,109]. Additionally, insulin clearance may be diminished in hypothyroidism coinciding with higher levels of counter-regulatory hormones, *i.e*., cortisol, glucagon, growth hormone and adrenalin [110]. Thus, despite increased gluconeogenesis in hyperthyroidism, and enhanced glucose-stimulated insulin secretion in overt hypothyroidism and SCH, plasma glucose levels tend to be higher in hypothyroidism [111,112]. Accordingly, a low-normal FT4 may relate to somewhat higher fasting plasma glucose levels [36].

Overt thyroid dysfunction is associated with profound effects on cardiovascular hemodynamics and cardiac function [1]. Triiodothyronine increases thermogenesis and decreases systemic vascular resistance, which in turn decreases effective arterial filling volume, stimulates renal reabsorption of sodium and increases blood volume [1]. An increased blood volume together with direct effects of triiodothyronine on the heart enhances cardiac inotropy and chronotropy, thereby stimulating cardiac output. In overt hypothyroidism, systemic vascular resistance is increased, whereas the heart rate and the ejection fraction are decreased, but the systemic hemodynamic effects of hypothyroidism are less outspoken than those accompanying hyperthyroidism [1]. SCH is associated with minor elevations in systolic and diastolic blood pressure. A meta-analysis comprising seven cross-sectional studies documented a small increase in systolic blood pressure of 1.89 mmHg and in diastolic blood pressure of 0.75 mmHg in SCH [113]. Accordingly, effects of low-normal thyroid function on systemic blood pressure are probably minimal. In the Busselton Health study and in the PREVEND study, blood pressure was not significantly associated with low-normal thyroid function [36,114]. In other surveys, positive associations of systolic or diastolic blood pressure with TSH were found [38,39], although positive associations of blood pressure with FT4 levels have also been reported [37].

## 4. Subclinical Hypothyroidism, Low-Normal Thyroid Function and Non-Alcohol Fatty Liver Disease

NAFLD includes a broad spectrum of pathology ranging from simple steatosis to non-alcoholic steatohepatitis (NASH), fibrosis and cirrhosis [115,116]. NAFLD also predisposes to hepatocellular carcinoma. NAFLD has become a leading cause of liver disease worldwide, and it is estimated that NAFLD occurs in more than 30% of American and European adults [117,118,119,120]. NAFLD is considered to reflect the hepatic component of MetS, since there is a strong association with insulin resistance, hypertension, obesity and dyslipidemia [121]. Accumulating evidence supports an association between NAFLD and increased risk of CVD [122,123].

Thyroid hormones play a key role in the hepatic lipid metabolism by a variety of mechanisms (see 2.2). Conversely, hepatic fat accumulation is regarded as the driving force of MetS- and T2DM-associated dyslipidemia [61,62,63]. These metabolic abnormalities may result in elevated plasma activity of phospholipid transfer protein, an emerging cardiometabolic risk factor which is intricately involved in HDL remodeling, triglyceride metabolism and anti-oxidant status [124,125,126]. Thyroid hormones do not only increase hepatic lipogenesis, but also enhance fatty acid β-oxidation [44,45,127]. Agonists of thyroid hormone receptor β, *i.e*., the subunit which is naturally expressed in hepatocytes, improve hepatic fat accumulation in animal studies [128]. Although increased fatty acid β-oxidation is anticipated to attenuate hepatic fat accumulation, this process may at the same time result in excessive mitochondrial production of reactive oxygen species. Among other mechanisms, thyroid hormones are likely to affect hepatic lipid accumulation and the subsequent development of fibrosis via an effect on the regulation of adiponectin which stimulates fatty acid oxidation and inhibits *de novo* lipogenesis [60,129,130]. Interestingly, genetic factors, such as variation in patatin-like phospholipase domain containing 3 (*PNPLA3*), which encodes a lipid droplet-associated, carbohydrate-regulated triglyceride hydrolase, probably play an important role in the development of NAFLD [131,132]. Little is currently known about gene-gene and gene-environment interactions that may be implicated in its pathogenesis [131].

A considerable number of studies have demonstrated an association between hypothyroidism and NAFLD. Subjects with hypothyroidism are about 1.5 to 2 times more likely to have biopsy-proven or ultrasonography confirmed NAFLD [59,133]. NAFLD is associated with hypothyroidism in a dose-dependent manner, independent of metabolic risk factors (SCH: odds ratio 1.36; overt hypothyroidism: odds ratio 1.71) [133]. Likewise, serum alanine aminotransferase (ALT) elevations, a surrogate marker of NAFLD [123], are associated with a higher TSH level across the spectrum of hypo- to hyperthyroidism [134]. In line, a systematic review suggested that NAFLD is related to hypothyroidism [60], although associations of NAFLD with thyroid function abnormalities have not been unequivocally reported [135].

There are a few studies which investigated the association of NAFLD with variations in thyroid function within the euthyroid range. Among 878 elderly Chinese subjects, NAFLD (prevalence 25.9%, determined by ultrasonography) was independently associated with lower FT4 levels [136]. Likewise, NAFLD (prevalence 26.5%, determined by ultrasonography) was associated with high-normal TSH and low-normal FT4 levels in another study in 739 Chinese subjects when taking account of metabolic risk factors [137]. In a large German study, an association of NAFLD (based on ultrasonorography and ALT elevations) with lower FT4, but not with lower FT3 or higher TSH levels was documented [138]. In another community-based Chinese survey study among euthyroid 1322 adults, TSH levels were higher in female subjects with NAFLD, but this difference disappeared after adjustment for adiposity [139]. In a small study in euthyroid subjects with biopsy-proven NAFLD, NASH was predicted by high-normal TSH levels [140]. In apparent disagreement with the above mentioned reports [133,134,136,137,138,139,140], NAFLD (determined by ultrasonorography) was more prevalent in subjects with low TSH level among 832 Iranian subjects, most of them being euthyroid, although FT4 levels were not different between subject with and without NAFLD [141]. In a study comprising 82 euthyroid subjects with and without MetS, we recently found that low-normal thyroid function, as judged from a higher TSH level within the normal range, may attenuate ALT elevations in the context of MetS and insulin resistance [142]. Taken together, it is plausible that NAFLD is associated with overt and subclinical hypothyroidism, but it is currently uncertain whether the same holds true for variations in thyroid function within the low normal range. Methodological issues with respect to the assessment of NAFLD, as well as ethnic differences in NAFLD susceptibility could in part explain the discrepancies.

## 5. Low-Normal Thyroid Function and Bilirubin

Bilirubin, the end product of heme catabolism, is a strong natural anti-oxidant, due to its ability to scavenge peroxyl radicals, a process which diminishes oxidative modification of LDL [143,144]. Besides anti-oxidant activity, bilirubin inhibits platelet activation, is able to attenuate expression of cellular adhesion molecules and has pro-atherogenic effects on lipoprotein metabolism [145,146,147,148]. Hence, the concept is emerging that bilirubin may play a role in the pathogenesis of cardiometabolic disorders characterized by increased oxidative stress and low-grade chronic inflammation [148,149]. In agreement, low circulating bilirubin levels relate to increased high sensitive C-reactive protein, as well as to serum amyloid A levels [150,151,152]. Low serum bilirubin probably attenuates the development of atherosclerotic manifestations [153,154,155,156,157,158], even independent from conventional risk factors [159].

The generation of bilirubin from heme involves conversion to biliverdin by the enzyme, heme oxygenase (HO), followed by reduction to unconjugated bilirubin by biliverdin reductase [144,148,149,150,151,152,153,154,155,156,157,158,159,160]. After diffusion into the circulation, where bilirubin is bound to albumin, bilirubin is taken up by hepatocytes, conjugated by uridine 5′-diphospho-glucuronosyltransferase (UGT1A1), and excreted into the bile. Two HO genes have been documented, of which the HO-1 enzyme is inducible, whereas HO-2 is constitutively expressed [149,161].

At least two pathways are involved in the regulation of bilirubin metabolism by thyroid hormones. First, HO-1 expression is induced by triiodothyronine, as documented by several *in vitro* models [162,163]. Second, bilirubin conjugation is controlled by thyroid hormone which decreases UGT1A1 enzymatic activity [164,165]. Since effects of thyroid hormone on these pathways are anticipated to increase circulating bilirubin, it is plausible to postulate that low-normal thyroid function may relate to lower bilirubin levels. To determine the extent to which low-normal thyroid function may influence serum bilirubin, we have recently carried out two studies among euthyroid subjects with varying degrees of glucose tolerance [166,167]. We first compared relationships of serum total bilirubin with thyroid function between non-diabetic and T2DM subjects [166], reasoning that alterations in bilirubin metabolism could affect enhanced oxidative stress which is a prominent feature of chronic hyperglycemia [168]. Serum bilirubin was related positively with FT4 in T2DM subjects, but not significantly in non-diabetic subjects [166]. To further delineate the impact of insulin resistance on the relationship of serum bilirubin with thyroid function, we explored whether more severe insulin resistance (homeostasis model assessment) modified the relationship of bilirubin with TSH in a large group of non-diabetic subjects [167]. In the whole population, there was a modest positive relationship of bilirubin with FT4. This relationship was more prominent in more insulin resistant individuals [167]. These results are consistent with the hypothesis that low-normal thyroid function may contribute to lower circulating bilirubin levels, and suggest that this effect is more outspoken in the context of chronic hyperglycemia and insulin resistance. Thus, it appears that low-normal thyroid function may affect bilirubin metabolism, thereby providing a potentially relevant mechanism contributing to enhanced oxidative stress and low-grade chronic inflammation with putative adverse consequences for atherosclerosis susceptibility.

## 6. Conclusions

In this review, we have delineated accumulating evidence which supports the concept that low-normal thyroid function, *i.e*., either higher TSH or lower FT4 levels within the euthyroid range, may play a pathogenetic role in the development of atherosclerotic cardiovascular disease. As yet, the extent to which low-normal thyroid function impacts on cardiovascular outcome is still unclear. It is also unknown whether possible adverse effects of low-normal thyroid function on cardiovascular outcome may be particularly relevant for specific populations, such as younger people and subjects at high cardiovascular risk. Alike subclinical hypothyroidism, low-normal thyroid function probably relates to modest increases in plasma total cholesterol, LDL cholesterol and triglycerides, but effects on HDL cholesterol are inconsistent. Additionally, low-normal thyroid function may be associated with insulin resistance and with other components of the metabolic syndrome, *i.e*., (central) obesity and plasma glucose. Effects of low-normal thyroid function on systemic blood pressure are minimal. Whereas the prevalence of NAFLD is increased in SCH, inconsistent effects of low-normal thyroid function on (markers of) NAFLD have been reported so far. Of further note, it has been recently demonstrated that low-normal thyroid function may promote plasma cholesteryl ester transfer protein-mediated cholesteryl ester transfer, and results in impaired ability of HDL to inhibit oxidative modification of LDL, both reflecting pro-atherogenic alterations in lipoprotein-associated processes. Low-normal thyroid function also relates to lower levels of bilirubin, which has strong anti-oxidative and anti-inflammatory properties. Remarkably, all these effects of low-normal thyroid functional status appear to be more outspoken in the context of chronic hyperglycemia and/or insulin resistance, which raises the possibility that diabetic subjects are particularly susceptible to consequences of low-normal thyroid function. Collectively, these findings agree with the concept that low-normal thyroid function may adversely affect several processes which conceivably contribute to the pathogenesis of atherosclerotic cardiovascular disease beyond effects on conventional lipoprotein measures.
